# Mitochondrial Regulation of Diabetic Kidney Disease

**DOI:** 10.3389/fmed.2021.745279

**Published:** 2021-09-27

**Authors:** Daniel L. Galvan, Koki Mise, Farhad R. Danesh

**Affiliations:** ^1^Section of Nephrology, The University of Texas at MD Anderson Cancer Center, Houston, TX, United States; ^2^Department of Nephrology, Rheumatology, Endocrinology and Metabolism, Okayama University Graduate School of Medicine, Dentistry and Pharmaceutical Sciences, Okayama, Japan; ^3^Department of Pharmacology and Chemical Biology, Baylor College of Medicine, Houston, TX, United States

**Keywords:** diabetic kidney disease, mitochondria, mitochondrial dynamics, oxidative phosphorylation, mitochondrial respiratory complexes, bioenergetics

## Abstract

The role and nature of mitochondrial dysfunction in diabetic kidney disease (DKD) has been extensively studied. Yet, the molecular drivers of mitochondrial remodeling in DKD are poorly understood. Diabetic kidney cells exhibit a cascade of mitochondrial dysfunction ranging from changes in mitochondrial morphology to significant alterations in mitochondrial biogenesis, biosynthetic, bioenergetics and production of reactive oxygen species (ROS). How these changes individually or in aggregate contribute to progression of DKD remain to be fully elucidated. Nevertheless, because of the remarkable progress in our basic understanding of the role of mitochondrial biology and its dysfunction in DKD, there is great excitement on future targeted therapies based on improving mitochondrial function in DKD. This review will highlight the latest advances in understanding the nature of mitochondria dysfunction and its role in progression of DKD, and the development of mitochondrial targets that could be potentially used to prevent its progression.

## Introduction

The kidney contains a great diversity of cell types in order to perform all of its endocrine and exocrine functions. Importantly, several different cell types in the kidney must act harmoniously in diverse microenvironments for the kidneys to function properly. An early indication as to the importance of mitochondria to the kidney function derives not only from their relative abundance in the kidney, but also the relative distribution of mitochondria specific to the needs and function of the cell type of the kidney with mitochondria-rich cells predominantly distributed in highly metabolically active proximal tubular cells, while podocytes and tubular epithelial cells of thin limb of Henle and collecting ducts exhibit comparatively a lower number of mitochondria (1–5).

**Figure 1:**
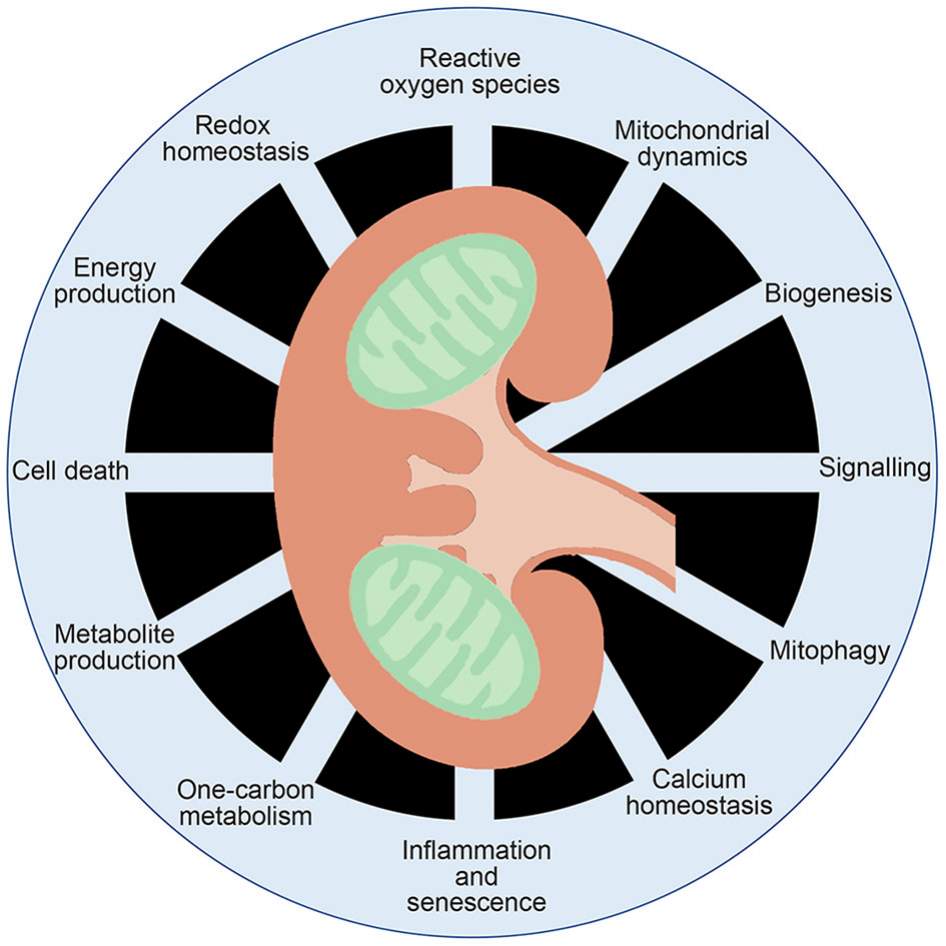
Multifaceted functions of mitochondrial function. Mitochondria are known to generate ATP and metabolites critical for signal transduction, as well as playing key roles in inflammation, calcium homeostasis, redox homeostasis and cell death.

Mitochondria are organelles with an endosymbiotic origin critical to proper function of eukaryotic cells. Central to the diverse functions of mitochondria are their bioenergetics properties serving as “powerhouses” of the cell generating adenosine triphosphate (ATP), as well as playing key roles in producing intermediates metabolites, reactive oxygen species (ROS) production, calcium homeostasis and apoptosis (Figure 1). As the most important physiological system for producing chemical energy stored as ATP from glucose, it is not surprising that mitochondria gained early attention as a possible target of diabetes and its micro/macrovascular complications.

The time course of mitochondrial dysfunction in the kidney has been documented in several experimental models of diabetic kidney disease (DKD) (6, 7). For instance, it was found that mitochondrial changes in size and function preceded histological and biochemical changes associated with kidney damage and these mitochondrial changes evolved with DKD progression (6). Indeed, altered mitochondrial morphology, bioenergetics and increased mitochondrial transition pore opening and ROS were all apparent prior to the presence of albuminuria (6, 8–12). These results suggest that mitochondrial dysfunction could be contributing to diabetic associated kidney damage.

Direct evidence that mitochondrial dysfunction can be a cause of chronic kidney disease (CKD) and DKD can also be gleaned by evaluating renal function in the presence of known mutations of mitochondrial associated proteins. The evidence is strengthened by several studies evaluating mutations in mitochondrial associated proteins that led to kidney dysfunction (13–21). Several independent mutations, relevant to mitochondrial function, result in kidney dysfunction, including prenyl diphosphate synthase subunit 2 (PDSS2) (22–24), mitochondrial inner membrane protein (Mpv17) (25), required for meiotic nuclear division 1 homology (RMND1) (26–30), ATP-binding cassette A1 (ABCA1) (12), apoptosis-inducing factor 1 (AIF1) (31), and several mitochondrial tRNAs (32–36). Podocyte-specific knockout of pdss2 further suggested the possible cell type specific consequences of some of these genes since it resulted in podocyte-associate renal disease. However, kidney damage was not apparent with conditional knockout of pdss2 in tubules, monocytes, or hepatocytes (22, 23). Podocyte knockout of ABCA1 was also shown to predispose the mice to DKD (12). Altogether, the evidence suggests that mitochondrial dysfunction can be a driving and primary cause of CKD and DKD, potentially playing an intrinsic and early role in disease progression. However, despite much interest, the precise nature of the changes to mitochondria and its physiological or pathophysiological significance remains elusive in DKD.

### Mitochondrial Function and DKD Progression

Due to the diverse pathways ascribed to mitochondria, there is not a single means to determine their function nor single biochemical assay to define their “health.” However, due to their classically assigned and pivotal role in energy production, many investigators have evaluated alterations in mitochondrial respiratory complexes, oxygen consumption rates, and/or ATP production as “proxy” for mitochondrial dysfunction with DKD progression. The oxygen consumption rate (OCR) measurements in the early phase of DKD (1–4 weeks after diabetic induction) in animal models indicated that metabolic activity was increased in renal cortex and proximal tubular cells (37–40), but subsequently declined with progression of albuminuria in experimental models of DKD (41). This would seem to be consistent with reports of increased respiratory complex activities in early phases of DKD (42, 43). However, other studies report contrasting results indicating decreased mitochondrial respiratory complex activities likely representing later stages of DKD (44–48). Similarly, while ATP levels within the kidney cortex have frequently been found unchanged during progression of DKD (49, 50), significantly lower levels of ATP have also been reported in other studies (6, 51). Our interpretation of these studies is that these results may indicate a compensatory increase in mitochondrial respiration early in DKD which is lost during progression of DKD.

The observations on mitochondrial function during DKD progression focusing mainly on tubular cells seem to be in contrast to the glomerular region of kidney cortex. Since glomerular cells are not as mitochondrial rich as tubules, the reduction in mitochondrial densities may allow for enhanced metabolic plasticity in these cells. Indeed, a number of studies seem to indicate that glomerulus and specifically podocytes have decreased OCR or metabolic activity from early onset of DKD which persist with progression of DKD (9, 52–54). In support of these observations, other reports suggest that mitochondrial respiratory complex activity is also decreased early on with DKD (45, 55, 56). The effects on ATP levels, however, are less clear. While several reports seem to indicate that ATP in podocytes is decreased (6, 9, 52), others have reported no major or little change (49, 50). These paradoxical results are not unexpected since podocytes have been reported to readily utilize glycolysis, possibly exhibiting a more flexible approach to ATP production (3). The inherent tissue differences in mitochondrial number and function highlights some of the limitations in our ability to complete a wholistic picture.

While mitochondria have been clearly demonstrated to be important players in the development and progression of DKD, the intricacies and nature of their dysfunction is not fully understood (57, 58). We and others have reported enhanced mitochondrial fission, increased mitochondrial ROS, and decreased oxidative phosphorylation (OXPHOS) in mouse models of DKD, whereas others have reported conflicting results. It is unclear if differing reports are due to different means of diabetic induction in animal models, the renal cell type examined, species-specific differences, or time of observation in the disease process. It will be an important future goal to reach a consensus on these questions. We will further highlight some of the current knowledge and possible gaps in defining the nature of mitochondrial dysfunction in DKD.

### Role of Mitochondrial Dynamics

Mitochondrial dynamics are the processes by which mitochondrial length, shape, and size are determined (59–61). Mitochondria have variable morphologies, even within the same cell, depending on the cell type, cellular needs and signaling cues. In its most basic and rudimentary understanding, mitochondrial morphology appears to be regulated by an ever changing and antagonistic intracellular balance between mitochondrial fission factors and mitochondrial fusion factors (62).

**Figure 2:**
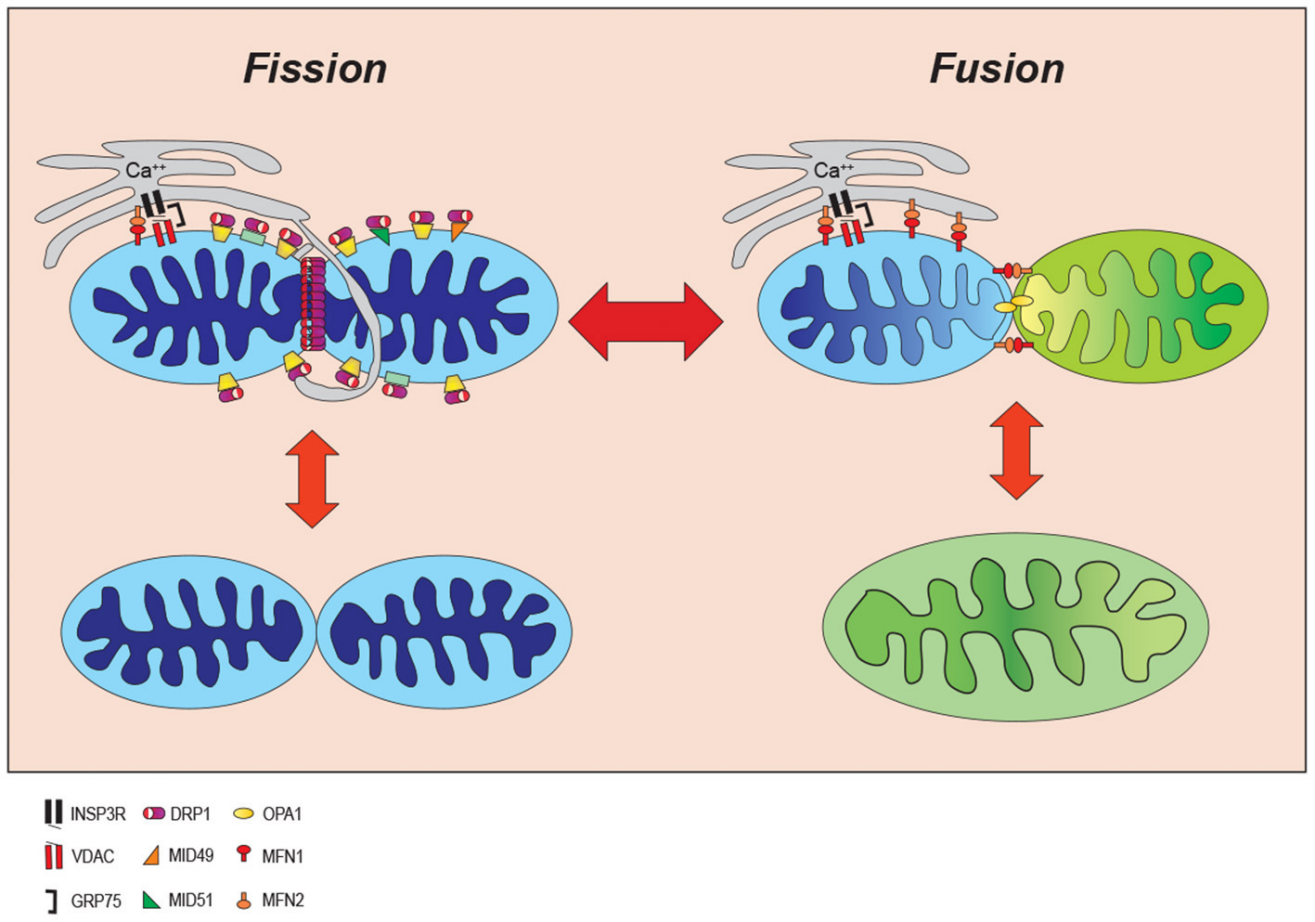
Mitochondrial dynamic. Mitochondria continuously change their size and shape by two opposing processes: mitochondrial fission and fusion. During the mitochondrial fission, mitochondria become fragmented in response to cell stress whereas they form an elongated shape increasing ATP production to adjust to cellular stresses. INSP3R, Inositol trisphosphate receptor; DRP1, dynamin-related protein 1; OPA1, optic atrophy 1; VDAC, voltage-dependent anion channel; MID49/51, mitochondrial dynamics proteins of 49 and 51kD; MFN1/2, mitofusin proteins 1 and 2; GRP75, glucose-regulated protein 75.

During mitochondrial fission, a mitochondrion is constricted to effectively divide a larger parent mitochondrion into smaller daughter organelles (59, 60). Mitochondrial fusion is the opposite process whereby smaller mitochondria have the outer and inner membranes joined to create a larger mitochondrion (59, 60). The balance between these factors of opposing action ultimately imparts characteristic size and shape of the mitochondria in a tissue-specific manner (Figure 2). Metabolic demands and signaling cues in a cell's microenvironment can push the balance toward mitochondrial fission to generate more fragmented and spherical mitochondria, or conversely toward mitochondrial fusion generating a more tubular and elongated morphology. Since this fluid process provides cells with rapidly responding metabolic flexibility, it is not surprising to realize that mitochondrial dynamics is highly regulated through a spatio-temporally precise cooperation among mitochondria, cytoskeleton, endoplasmic reticulum, and resident and recruited mitochondrial-associated proteins (11, 63–67).

While mitochondrial fission can be viewed as a process of sequential discrete steps, the order and independence of each step remains to be fully understood. An early step is marking of the site where the mitochondria will divide. The current model suggests that the endoplasmic reticulum (ER) initially marks fission furrows in the mitochondria where mitochondrial fission will ultimately occur (65, 66, 68). Increases in cytoplasmic calcium drive actin assembly around the ER protein inverted formin 2 (INF2) and the actin polymerization is believed to provide some force for constriction (64, 67). The association of INF2 with mitochondrial localized Spire 1C, links the mitochondrial, actin polymerization event between the two organelles (mitochondria and ER) (69) and will enhance calcium transfer from ER to mitochondria via mitochondrial calcium uniporter 1 (MCU1) initiating constriction of the mitochondrial inner membrane prior to outer membrane constriction in a process which requires activation of the electron transport complexes and the mitochondrial metalloendopeptidase, OMA1 (70). Mitochondrial fission will further proceed by recruitment of the cytoplasmic fission factor, dynamin-related protein 1 (DRP1) to the outer mitochondrial membrane (71–74). DRP1 is recruited to the mitochondrial outer membrane where it oligomerizes to form a ring around the mitochondria at the fission furrow. DRP1 is anchored to the mitochondria by interactions with its mitochondrial receptors including mitochondrial fission 1 (FIS1), mitochondrial fission factor (MFF), and mitochondrial dynamics proteins of 49 and 51 kD (MiD49/MiD51). Constriction of the mitochondrial membrane utilizes DRP1-driven GTP-hydrolysis for energy to drive mitochondrial fission.

DRP1 translocation to the mitochondria is further regulated by several posttranslational modifications including phosphorylation (52, 75–80), *O*-GlcNAcetylation (81), sumoylation (82–84), and S-nitrosylation (85–87). DRP1 activation is also enhanced by binding with actin (88), actin-related proteins (11, 89), AKAP1 (80), cardiolipin, and palmitic acid (90–92).

Mitochondrial fusion, on the other hand, is mediated by another dynamin related protein, optic atrophy 1 (OPA1), at the mitochondrial inner membrane and mitofusin proteins 1 and 2 (MFN1 and 2) at the outer mitochondrial membrane. MFN1/2 can interact both as homo- and hetero-dimers to mediate fusion of the outer mitochondrial membrane. OPA1 appears to be regulated in part by post-translational changes driven by the mitochondrial membrane potential and interactions with the mitochondrial OMA1 zinc metallopeptidase. In addition, to its function in mitochondrial fusion, OPA1 also plays a key role in maintaining mitochondrial cristae morphology and respiratory ETC function by sequestering cytochrome c within the cristae. The importance of these mitochondrial dynamic protein factors to life is evidenced by findings that knockout of several member proteins is embryologically lethal (93–96).

Enhanced mitochondrial fission is reported in multiple cell types of the kidney including tubules and podocytes in animal models of DKD (1, 11, 52, 97–103). In support of these preclinical studies, clinical evidence have revealed increased fragmented mitochondria in several cell types within the kidney cortex of diabetic patients as well (99, 104, 105). Our studies in the *db/db* model of DKD identified enhanced mitochondrial fission and increased expression of DRP1 in both glomerular endothelial cells and podocytes (8). Importantly, while podocyte-specific depletion of DRP1 had no effect on mitochondrial function, DRP1 deficiency specifically in podocytes in diabetic *db/db* mice improved DKD progression by improving mitochondrial function suggesting a role for cellular stress to unravel the effect of DRP1 on mitochondrial function (52). The tendency toward mitochondrially fragmented morphology has been tied most strongly to several proteins regulating mitochondrial fission (11, 52, 74, 80, 100, 106, 107). Other studies have confirmed these initial observations in other models of DKD. For example, Myo-inositol oxygenase (MIOX) expression was shown to be increased in kidneys of *db/db* mice and streptozotocin (STZ)-treated diabetic mice contributing to progression of DKD, and linked to enhanced DRP1 and FIS1 expression with decreased MFN2 expression (98, 108). The Src homologous-collagen homolog adaptor protein, p66Shc, expression and phosphorylation were also increased in kidneys of both *db/db* mice and STZ-treated diabetic mice, and were found to correlate with increased DRP1 and FIS1 expression and decreased MFN1 expression (99, 109). Knockdown of *Fis1* prevented mitochondrial fragmentation, restored MFN1 expression, and reduced p66Src binding to FIS1 under high glucose conditions (99). Dual-specificity protein phosphatase−1 (DUSP1) was shown to be decreased and JNK pathway activation increased in the kidneys of STZ diabetic mice and linked to increased DRP1 and MFF expression with decreased MFN1 and OPA1 expression (101). Finally, the expression of hypoxia inducible factor 1 (HIF1) was conditionally deleted in proximal tubular cells of STZ treated diabetic mice showed enhanced DKD progression with increased expression of DRP1 and FIS1 with decreased MFN1 expression. *In vitro* it was suggested that HIF1 modulates these changes by its target heme oxygenase-1 (HO-1) (110).

Post-translational modifications of DRP1 and specifically its phosphorylation also seem to play a critical role in pathogenesis of DKD. We and others have found that DRP1 phosphorylation at the human residue S637 and equivalent mouse residue serine 600 (S600 in mouse DRP1 isoform b), hereafter referred to as S600, enhances DRP1 activity and translocation to the mitochondria to mediate enhanced mitochondrial fragmentation (8). We have shown that Rho-associated, coiled-coil containing protein kinase 1 (ROCK1) activation in the diabetic kidney phosphorylates DRP1 at S600 both *in vivo* and *in vitro* triggering mitochondrial fragmentation (8). Recently, it was shown that S600 of DRP1 in renal tubules maybe phosphorylated by the compartment directing, A kinase (PRKA) anchor protein 1 (AKAP1), localizing protein kinase A (PKA) to the outer mitochondrial membrane and triggering mitochondrial fragmentation in a STZ model of type-1 diabetes (80). We also provided *in vivo* evidence indicating that knock-in diabetic *db/db* mice mutating S600 in DRP1 to the non-phosphorylable alanine at position 600 (S600A) exhibited marked improvement in DKD progression and protected mitochondrial morphology and bioenergetics of podocytes. Mechanistically, it was shown that phosphorylation of DRP1 at S600 enhanced its interaction with both MFF and the actin related protein 2/3 complex (ARP2/3) enhancing mitochondrial localization of DRP1 and triggering mitochondrial fission (11). Similarly, it has been reported that phosphorylated DRP1 was increased and the expression of MFN1 markedly decreased in proximal tubular cells isolated from *db/db* mice, while treatment of diabetic mice with a β2-agonist, formoterol, decreased phosphorylated DRP1 levels and restores MFN1 levels (51).

While there is a growing body of evidence indicating that mitochondrial fission is a key morphological indicator of kidney damage in DKD, hyperfused and large mitochondria may also have a role in DKD progression (111). Hyper-elongated mitochondria may be an indicator of cellular senescence and associated with mitochondrial DNA damage, loss of mitochondrial membrane potential, and enhanced ROS as well (112–115).

Overall, the evidence seems to indicate that renal damage in DKD is associated with a shift in mitochondrial dynamics toward enhanced fission. The evidence is clear that DRP1 lies at the center of this dynamic and has been found to be increased and/or modified in multiple kidney cell types. These changes are frequently found in conjunction with increased expression of fission proteins such as FIS1 and MFF and decreased expression of MFN1. The functional consequences of tipping the mitochondrial dynamic balance toward fission seem to share deleterious end points such as enhanced ROS contributing to DKD progression.

### Mitochondrial Bioenergetics and Oxidative Stress

Cellular ATP is maintained through two interconnected metabolic pathways, glycolysis and oxidative phosphorylation (OXPHOS). During glycolysis, glucose is transported into the cell cytoplasm and converted into 2 molecules of pyruvate to generate 2 ATP molecules. In the absence of oxygen, glycolysis will anaerobically ferment the pyruvate to lactate generating 2 NADH in the cytoplasm. However, in the presence of oxygen, pyruvate will be decarboxylated into acetyl coenzyme A (AcCoA) inside mitochondrial matrix and enter the tricarboxylic acid (TCA) cycle. The TCA cycle is an enzymatically controlled series of oxidation steps culminating in production of CO_2_ and 8 NADH, 2 FADH_2_, and 2 ATP molecules. Ultimately OXPHOS can harvest 30–36 ATP from the entry of the NADH and FADH_2_ per glucose depending upon the amount of proton leak.

**Figure 3:**
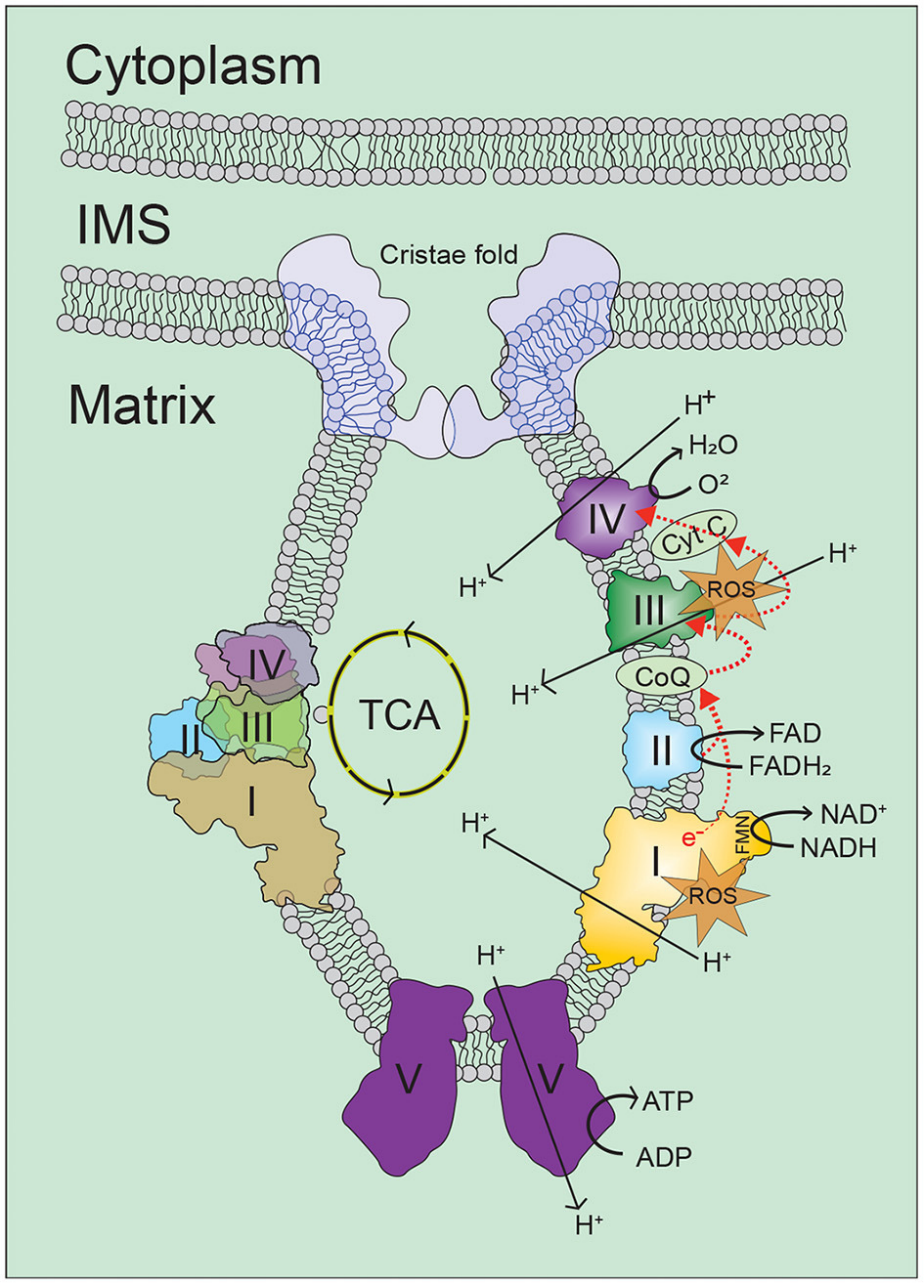
Oxidative phosphorylation. Cellular energy in the form of ATP is mainly generated in mitochondria by the oxidative phosphorylation (OXPHOS) process, in which electrons on the inner-membrane of the mitochondria are passed through a series of mitochondrial complexes (Complexes I-V) in redox reactions. Energy released in these reactions is then coupled to ATP generation. Increase in intracellular levels of NADH and FADH2 drives oxidative phosphorylation, which leads to increase of oxygen consumption and ATP production by ATP synthesis. For more information, refer to the main text. ROS, reactive oxygen species; TCA, tricarboxylic acid; I/II/III/IV/V, mitochondrial respiratory complex I/II/III/IV/V; NADH, reduced nicotinamide adenine dinucleotide; NAD, nicotinamide adenine dinucleotide; FADH_2_, reduced flavin adenine dinucleotide; FAD, flavin adenine dinucleotide; FMN, flavin mononucleotide; CoQ, coenzyme Q; Cyt C, cytochrome C.

OXPHOS is comprised of 4 respiratory complexes (I-IV) within the inner mitochondrial membrane which are collectively referred to as the electron transport chain (ETC) in which a series of redox reactions are converted into a proton motive force by pumping protons into the mitochondrial intermembrane space (Figure 3). Complex I accepts electrons from NADH while complex II accepts electrons from FADH_2_ and both transfer the electron to coenzyme Q (CoQ). Complex III in conjunction with cytochrome c can accept these electrons and pass them to complex IV with oxygen as the terminal electron acceptor. Complex V or ATP synthase allows passage of protons back to the matrix linked to generation of ATP. Electron escape during ETC reactions is capable of generating ROS which under physiological conditions are both quenched by endogenous antioxidant mechanisms and utilized as important cellular signaling molecules. It has been suggested that increased ROS generation and decreased ROS quenching result in oxidative damage to cellular components and mitochondria capable of resulting in cell death. This apparent paradox may exist with low levels of ROS serving as survival signaling during conditions of stress while once reaching a threshold become damaging to the cell and synergistically contribute to enhanced mitochondrial dysfunction.

Superoxide production during ETC transport was first reported in 1966 (116), and has been an area of interest ever since. Complex I (117–120) and complex III (118, 121–124) are believed to be the principle sites of mitochondrial ROS generation during ETC transport, of which complex I is believed to produce the majority of mitochondrial ROS (122, 125–127). Complex III can produce both intermembranous and matrix superoxide during transport of electrons through the quinol (Q)-cycle depending on the mitochondrial membrane potential and oxidation state of cytochrome c (122, 123). Complex I can produce superoxide by two distinguishable mechanisms. When the NADH/NAD^+^ ratio is high and respiratory chain activity is inhibited, the matrix facing flavin mononucleotide (FMN) site can produce superoxide (119, 128–130). Alternatively, superoxide can be generated when mitochondrial potential drives reverse electron transport at complex I. Reverse transport occurs when the mitochondrial potential is high and CoQ is reduced forcing the reduction of NAD to NADH at the flavin mononucleotide (FMN) site (121, 131–133). In DKD, it has been demonstrated that both complex I (134) and complex III (135) can generate superoxide and increased mitochondrial ROS in the kidney (136–138). Transgenic expression of superoxide dismutase or thioredoxin protected the kidney in mouse models of DKD (139, 140). However, not all antioxidants were equally effective as transgenic glutathione peroxidase-1 expression in STZ-treated mice did not have renal protection (141).

Substantial evidence has accumulated in patients and animal models of DKD indicating that mitochondrial ROS is significantly increased in the kidney and generated the free radical theory of diabetic microvascular complications (142–144). The “Unifying Hypothesis” suggests that chronically driven glucose over production of mitochondrial ROS at the mitochondria leads to cellular and eventual end kidney failure. Increased mitochondrial ROS production has been demonstrated both *in vitro* and *in vivo* in multiple mouse models of DKD (39, 41, 56, 100, 109, 145–148). However, an important gap in our current understanding of the role of mitochondrial ROS in DKD pathogenesis is to identify the source(s) of enhanced mitochondrial ROS in DKD. The increased mitochondrial ROS production was initially proposed has been proposed to be linked to mitochondrial dynamics remodeling and biogenesis (149). This suggestion was supported experimentally by some recent studies indicating that overexpression of DRP1 or MFF, as well as knockdown of MFN1/2 together or alone in cultured cells, lead to mitochondrial fragmentation and increased mitochondrial ROS (149–154). Increased expression of p66Shc, NR4A1, ROCK1/DRP1, and HIF1 (hypoxia inducible factor 1) in DKD also caused fragmented mitochondria and increased mitochondrial ROS and apoptosis (8, 98, 99, 101, 110, 155). Decreased expression of DUSP1, MIOX, or PGC1α in the DKD were similarly reported to increase mitochondrial ROS and apoptosis (98, 101, 145). Increased production of mitochondrial ROS appears to be a central effector of cellular damage, but is inherently difficult to measure mitochondrial ROS *in vivo* due to their multiple species and frequently very short biological half-lives. Indeed, a central challenge in addressing the role of redox biology in DKD progression is to accurately measure mitochondrial ROS. Importantly, studies addressing mitochondrial ROS have resulted in conflicting interpretations mainly because of variations in the detection methods employed with a wide range of experimental approaches, including the use of fluorescent indicators of ROS, electron paramagnetic resonance (EPR), spectrophotometry, and high-performance liquid chromatography (HPLC); each method with its own limitations and advantages and generally specific to the ROS molecule attempting to be measured, cross reactivity, cellular permeability and localization.

We have recently used a transgenic, redox-sensitive GFP based biosensor specifically expressed in the mitochondrial matrix to determine mitochondrial generated ROS in a *db/db* mouse model *in vivo* (56). Kidney from transgenic control and diabetic mice were examined by 2-photon microscopy followed by ratio-metric determination of the redox state of the biosensor. Increased mitochondrial ROS in the diabetic kidneys was found which strongly implicated complex I as a key generator as the biosensor was matrix localized and the increase in ROS was prevented by a genetic bypass of complex I. This report and others have utilized mitochondrial targeted antioxidants such as mitoTEMPO, elamipretide, and others to demonstrated reduced mitochondrial ROS correlating with improved histological features of DKD in mouse models (12, 56, 156, 157).

Evidence determining mitochondrial ROS in the kidney of diabetic mice has also been obtained using dihydroethidium (DHE) as the redox sensor (45). Results in these mice were in contrast to the previous studies describing decreased mitochondrial ROS in the diabetic kidney. However, both studies were in agreement in regards to decreased activity of mitochondrial respiratory chain activity and found evidence of oxidative stress in the kidneys of diabetic animals (45, 56). These contrasting findings might be indicative of the difficulty in interpretating the cross-talk among different sources of ROS production (45, 56, 100). One such point of cross-talk in DKD could be derived from NADPH oxidases pathway. The NADPH oxidases of the NOX family are important enzymatic sources of ROS whose main biological function is electron transport across the plasma membrane and generate ROS by reducing oxygen to superoxide and/or hydrogen peroxide (158). At least seven homologs of NOX are present in the human genome: NOX1 to NOX5, DUOX1, and DUOX2. These mainly differ in their activation mechanisms, tissue distribution, and type of ROS production (157). Among different members of NOX family, NOX4 expression has been shown to be increased in the kidneys of diabetic mouse models, and capable of producing different types of ROS, mainly hydrogen peroxide (45, 159–163). However, under stress conditions, NOX4 might be translocated to mitochondria contributing to enhanced mitochondrial ROS by regulating mitochondrial respiratory complex I activity (164, 165). Consistent with this notion, deletion and pharmacological inhibition of NOX4 have been demonstrated to attenuate progression of DKD (161). NOX5 is also increased in the human diabetic kidney but not encoded by the mouse genome. Nevertheless, it has been shown that forced ectopic expression of NOX5 in mouse models leads to accelerated progression of DKD which could be ameliorated by pan-NOX inhibitors (161, 166–168).

While the specific contribution of mitochondrial generated superoxide remains an open question, it is clear that there is enhanced ROS in the kidneys of diabetic mouse models probably arising from multiple sources. The generated ROS is usually carefully balanced to stimulate stress abrogation responses, while not exceeding the cell ability to protect itself from damage through anti-oxidant enzymes. Once the balance is shifted such that the production of ROS exceeds the cells inherent antioxidant protections, an increasing cycle of cell damage is elicited resulting in compromised mitochondrial function, damaged mitochondrial DNA and proteins (169). If the cell cannot re-establish its balance, the end result is cellular death and kidney dysfunction.

### Mitochondrial Biogenesis and Mitophagy

Mitochondrial biogenesis and degradation are highly regulated in order to maintain a healthy pool of mitochondria in the cell. However, both of the processes are dysregulated in DKD. Mitochondrial biogenesis refers to the cellular regulation of mitochondrial abundance titrated through an interconnected set of transcription factors. Central among these transcription factors is the peroxisome proliferator-activated receptor gamma (PPAR) coactivator-1 family of transcriptional coactivators (PGC1α/β) and PGC-1-related coactivator (PRC), coined as “master regulators” of mitochondrial biogenesis.

PGC1α was initially identified by the Spiegelman group (170) as a binding partner of PPAR that is highly expressed in tissues with high energy demand such as the kidney. As a coactivator, PGC1 does not bind to DNA promoters directly, but in dimerization with a variety of transcription factors to modulate a series of mitochondrial active gene products (171, 172). A few of the better understood partners of PGC1α include nuclear respiratory factor 1 (NRF1), NRF2, and the estrogen-related receptors (ERR). These heteromeric dimers likely, at least in part, could explain why experimental results with modulating PGC1α appear so highly tissue-specific since the possible dimeric combinations and relative amounts could depend on a specific tissue's expression levels of PGC1α, its various binding partners, and posttranslational modifications. Regardless, the system allows for a high degree of specialization in the regulation of gene products impinging upon mitochondrial biogenesis, mitochondrial gene transcription, fatty acid oxidation, TCA cycle, and OXPHOS. The role of PGC1α as a transcriptional rheostat tuning metabolic cellular function to physiological energy demands has been experimentally demonstrated in a myriad of tissues.

A number of studies have provided strong evidence that decreased PGC1α and reduced mitochondrial biogenesis are key features in the development of DKD. PGC1α has been demonstrated to be significantly decreased in the diabetic kidneys (9, 45, 145, 173–175). STZ treated rats have decreased PGC1α in renal tubules. This is evident in several mouse models of DKD as well. Diabetic OVE26, AKT2, and *db/db* mouse models have all been illustrated to have decreased PGC1α in the kidneys (176, 177). PGC1α was demonstrated to play a key role in another study examining an enzyme believed to couple glycolysis to mitochondria bioenergetics, pyruvate kinase M2 (PKM2) (10). In this study, podocyte-specific depletion of PKM2 in diabetic mice exacerbated diabetic renal injury, while pharmacological activation of PKM2 protected diabetic mice from kidney damage. Importantly, increased levels of PKM2 were correlated to protection from DKD in diabetic patients. The underlying mechanism proposed was that the protection was due in part to PKM2 linked activation of PGC1α and improved mitochondrial function (10, 178).

Our group has demonstrated that PGC1α could also be regulated by a long non-coding RNA, Tug1 (taurine upregulated 1). We found that Tug1 overexpression protects *db/db* mice from DKD (9). The protection was linked *in vitro* to Tug1 binding to PGC1α and improved mitochondrial function. However, another report found that podocyte-specific inducible overexpression of PGC1α in mouse models of DKD failed to offer renal protection (179). High expression levels of PGC1a could potentially drive a mitochondrial substrate preference toward b-oxidation of lipids contributing to worsening phenotype of DKD in experimental models. These results may indicate that PGC1α levels must be regulated and maintained within a very limited range to be beneficial.

PGC1α offers renal protection, at least in part, by driving oxidized nicotinamide adenine dinucleotide (NAD+) biosynthesis (180, 181). The redox imbalance of NADH/NAD^+^ (reduced/oxidized) is high in the diabetic kidney as electrons from the breakdown of nutrients become stored as NADH and metabolic pathways such as sirtuins consume NAD^+^. Complex I or lactate dehydrogenase can then regenerate NAD^+^ through oxidation of NADH (182–185). Modulation and the end balance of these processes determine the NADH/NAD^+^ ratio and represents one intersection point of PGC1α with sirtuins in mitochondrial biogenesis and bioenergetics.

The family of NAD-dependent deacetylases known as Sirtuins (SIRT1-7) regulate mitochondrial biogenesis and function as a nutritional rheostat which effects mitochondrial function via protein acetylation and have been implicated in several pathologies, including DKD (186–189). Proximal tubular overexpression of SIRT1 protected diabetic mice from DKD. Knockout of SIRT1 exacerbated renal injury in two separate diabetic mouse models and induced albuminuria in non-diabetic animals (190). The SIRT1 agonist, resveratrol, reduced podocyte damage in diabetic mice by activating PGC1α as well as its targets NRF1 and mitochondrial transcription factor 1 (TFAM) to improve mitochondrial function and reduce oxidative stress. SIRT1 has been shown to play a protective role in both tubules and podocytes of diabetic mouse models. The renoprotection stems in part through deacetylation of transcription factors, including PGC1α and PPARy (181, 191, 192). Podocyte-specific overexpression of SIRT1, and several non-specific agonists of SIRT1 such puerarin have been shown to attenuate DKD in animal models (193, 194). A more specific agonist, BF175, was tested and was shown to protect the kidney in type 1 diabetic OVE26 mice (195).

Consistent with the interplay between PGC1α and SIRT1, it has been shown that PGC1α can also increase levels of the mitochondrially-localized, SIRT3 (175, 196, 197). SIRT3 has been demonstrated to regulate mitochondrial function through direct binding to ETC proteins, mitochondrial dynamics, redox protection, and TCA cycle modulation and is the main mitochondrial deacetylase regulating cellular ROS. The SIRT3 agonist, honokol, was tested in BTBR *ob/ob* mice with type 2 diabetes and determined to be protective in DKD (186). SIRT3 was determined to be significantly decreased in the kidney of BTBR *ob/ob* mice in conjunction with increased ROS levels. Treatment with Honokol, a Magnolia tree bark extract and SIRT3 activator, reduced albuminuria and podocyte injury in the diabetic mice and was found to restore PGC1α levels in glomerular cells. The protective role of SIRT3 on glomeruli was mediated in part through increased SIRT3 tubular expression and upregulation of tubular nicotinamide phosphoribosyl transferase (Nampt), suggesting a possible tubule-glomerulus retrograde signaling mechanism. The lack of regulation of SIRT3 in glomeruli and postulated tubular-glomerular signaling was also a finding of a study examining SIRT1 in the diabetic kidney (190) where diabetic glomerular damage was improved by selective upregulation of tubular SIRT1.

In contrast to mitochondrial biogenesis, the process of mitophagy is the physiological clearance mechanism for removal of damaged mitochondria from the cell which appears to become overwhelmed in DKD (198–200). Mitophagy appears to have both a ubiquitin-dependent and -independent pathway (201, 202). The ubiquitin-dependent pathway is dependent upon mitochondrial dynamics, energetics, transport, and autophagic factors. The phosphatase and tensin homolog (PTEN) induced putative kinase 1 (PINK1) and Parkin (PRKN) are key mediators of the pathway. Physiologically PINK1 is transported to the inner mitochondrial membrane and proteolytically degraded in a ubiquitin dependent manner. When mitochondria become damaged and depolarized PINK1 is autophosphorylated and stabilized on the outer mitochondrial membrane to recruit PRKN and its E3 ligase activity. Mitochondrial fate is determined by the balance of the ubiquitination/deubiquitination process whereby increased poly-Ub targets the mitochondrion for proteasome destruction. PINK1 can increase mitochondrial fission by indirectly increasing DRP1 activity while the PINK1/PRKN interaction enhances Mfn2 degradation (203–210). Ubiquitin-independent pathway involves several ubiquitin E3 ligases which can localize to mitochondria and recruit autophagic factors.

The kidney has been shown to have a high rate of mitophagy relative to other organs, as well as cell type dependent regulation where podocytes have greater levels of mitophagy relative to tubules (211, 212). Increased mitophagy has been shown to be protective in models of CKD, DKD, and AKI (213–220). The PINK1/PRKN pathway is activated by oxidative stress established in DKD whereas treatment with the mitochondrial antioxidant, MitoQ, has been suggested to protect from DKD by increasing mitophagy levels (218). Tissue-specific knockout of ATG5 in a STZ model of DKD revealed that podocyte deletion induced podocytopathy and glomerulosclerosis while endothelial-specific knockout accelerated progression of DKD and when deleted in both tissues together increased DKD (221). These observations in aggregate suggest a critical role of mitophagy in DKD progression.

## Conclusion and Future Perspectives

In this review, we touched the surface of several possibilities by which mitochondrial dysfunction could contribute to the development and progression of DKD, but we recognize that there still much remains to be uncovered. We would like to underscore a few gaps in knowledge for future discoveries.

As of yet, it is difficult to reach a clear consensus on the time course of mitochondrial respiratory activity and OXPHOS changes during progression of DKD. We await the arrival of more specific bioreporters to evaluate specific sources of enhanced ROS in real time in living animals, which ideally could link ROS to their enzymatic source in mitochondria. Similarly, a complete understanding of how mitochondrial dynamics fidelity is regulated and an evaluation of the “coincident detection” to fully integrate multiple organelles and biological factors into a single framework remains to be fully accomplished. The mitochondrial biogenesis pathway, and PGC1α in particular, are attractive therapeutic targets for DKD, but likely await the ability of targeting this pathway selectively in the kidney within a narrow therapeutic window. Finally, mitophagy, the crossroads of diverse signaling pathways, has shown a great promise as a therapeutic target, but the molecular mechanisms by which mitochondrial packaging for mitophagy becomes uncoupled during DKD progression remains unclear.

In conclusion, there have been increasing efforts to better define the nature of mitochondrial dysfunction in DKD over the past two decades. Initial studies utilizing metabolic screening approaches to identify the best possible biomarkers for predicting DKD susceptibility and progression are currently on-going (222, 223). While these and other studies have identified several mitochondrially-derived molecules such as mitochondrial DNA in serum and/or urine as potentially useful markers for DKD progression, none has exceeded expectations and are not currently available for patients care. Looking forward, opportunities in mitochondrial medicine involve the use of “multi-omics” and proteogenomics to provide further insights into the role of mitochondrial biomarkers in predicting DKD progression. A quantitative assessment of mitochondrial dysfunction in patients with DKD could accelerate the identification and development of novel biomarkers and treatments, and improve the ability to assess the efficiency of new drugs by measuring mitochondrial function pre- and post-therapies. Finally, the genetic and hormonal environment of the male and female kidney is significantly different, and these differences have been implicated on the onset and progression of DKD in both Type1 and 2 diabetes (224–227). The impact of gender on mitochondrial bioenergetics and function in kidney diseases has recently been reported (228). While many questions still remain to be carefully addressed, it seems clear that sexually determined differences in mitochondrial biogenesis, bioenergetics, and ROS generation exist, and these differences may also contribute to differences in long-term prognosis in patients with DKD (229, 230). Further research is needed to conclude a causal association between differences in gender, mitochondrial dysfunction and progression of kidney disease in large diabetic population.

## Author Contributions

DG is responsible for writing the manuscript and literature research. KM and FD reviewed the manuscript and made significant revisions on the drafts. All authors have read and agreed to the final version of the manuscript.

## Funding

This study was supported by grants from the National Institutes of Health RO1DK078900 (FD) and RO1DK091310 (FD).

## Conflict of Interest

The authors declare that the research was conducted in the absence of any commercial or financial relationships that could be construed as a potential conflict of interest.

## Publisher's Note

All claims expressed in this article are solely those of the authors and do not necessarily represent those of their affiliated organizations, or those of the publisher, the editors and the reviewers. Any product that may be evaluated in this article, or claim that may be made by its manufacturer, is not guaranteed or endorsed by the publisher.
